# Carbazole- and Triphenylamine-Substituted Pyrimidines: Synthesis and Photophysical Properties

**DOI:** 10.3390/molecules24091742

**Published:** 2019-05-05

**Authors:** Sylvain Achelle, Julián Rodríguez-López, Massinissa Larbani, Rodrigo Plaza-Pedroche, Françoise Robin-le Guen

**Affiliations:** 1 Université de Rennes, CNRS, Institut des Sciences Chimiques de Rennes-UMR 6226, F 35000 Rennes, France; massilarb06@gmail.com (M.L.); francoise.le-guen@univ-rennes1.fr (F.R.-l.G.); 2 Área de Química Orgánica, Facultad de Ciencias y Tecnologías Químicas, Universidad de Castilla-La-Mancha, Avda. Camillo José Cela 10, 13071 Ciudad Real, Spain; rodripedroche@gmail.com

**Keywords:** pyrimidines, fluorescence, white-light emission, intramolecular charge transfer

## Abstract

A series of pyrimidine derivatives bearing one, two or three triphenylamine/9-ethylcarbazole substituents has been synthesized by Suzuki cross-coupling reaction. All compounds showed absorption bands in the UV region and the emission of violet-blue light upon irradiation. Protonation led to quenching of the fluorescence, although some derivatives remained luminescent with the appearance of a new red-shifted band in the spectra. Accurate control of the amount of acid enabled white photoluminescence to be obtained both in solution and in solid state.

## 1. Introduction

The pyrimidine (1,3-diazine) core is a π-deficient six-membered heterocycle with two nitrogen atoms. Consequently, the pyrimidin-4-yl and the pyrimidin-2-yl fragments act as relatively strong electron-withdrawing groups [1]. With regard to pyridyl analogues, the presence of an appropriately positioned second nitrogen atom significantly enhances their electron-attracting character [2]. The pyrimidine ring has therefore been extensively used as an acceptor unit in push-pull structures in which significant intramolecular charge transfer (ICT) occurs [1,3,4]. In this respect, 4,6-di(arylvinyl)pyrimidines are now well established two-photon absorption chromophores for biological imaging [5,6,7,8], 3D lithographic microfabrication [9], and 3D data storage [10]. Appropriately substituted 2,4,6-triarylpyrimidines have also been developed as efficient emitters for OLEDs due to their thermally activated delayed fluorescence (TADF) properties [11,12,13,14,15]. Pyrimidine push-pull chromophores have also been developed as second order nonlinear optic (NLO) materials [16,17] and as dyes for photovoltaic applications [18,19]. 

When compared to their arylvinyl- and arylethynyl- analogues, arylpyrimidines generally exhibit a blue-shifted emission with a higher emission quantum yield [1,20]. Arylpyrimidines can be easily obtained by Suzuki cross-coupling reaction from halogenopyrimidines [21,22,23,24]. The electron-withdrawing character of the pyrimidinyl fragments allows this reaction to be performed from chloro-derivatives [25]. 

The emission properties of pyrimidine fluorophores are highly sensitive to the environment. As a consequence, a strong emission solvatochromism, which is typical of ICT chromophores [26,27,28,29], is observed: a polarity increase provides a significant bathochromic shift of the emission band [30,31,32,33]. On the other hand, protonation of the pyrimidine ring (pK_a1_ = 1.1) significantly enhances its electron-withdrawing character and leads to a red-shift of both absorption and emission bands. These shifts are generally accompanied by emission quenching, but in some cases, particularly with weak electron-donating groups such as methoxy fragments, red-shifted emission with increased intensity is observed [32,34,35]. White photoluminescence can be obtained both in solution and in doped polystyrene films when the neutral and protonated forms are present in the appropriate ratio [34,35].

Triphenylamine and 9-ethylcarbazole have been extensively used as electron-donating units in push-pull structures [36,37,38,39]. Although these units are weaker electron-donors than *N*,*N*-dialkylanilines [40], derivatives that incorporate these moieties generally have stronger luminescence properties [41,42,43]. Nevertheless, only a few pyrimidine derivatives bearing triphenylamine or 9-ethylcarbazole fragments have been described in the literature [9,19,44,45]. 

We report here the synthesis of a series of pyrimidine chromophores with one, two or three triphenylamine/9-ethylcarbazole substituents. The photophysical properties of the new materials were carefully studied and the luminescence behaviour in the presence of acid was evaluated. In some cases, the protonated pyrimidines remained emissive, which enabled white luminescence to be obtained by accurate control of the amount of acid.

## 2. Results and Discussion

### 2.1. Synthesis

**Scheme 1:**
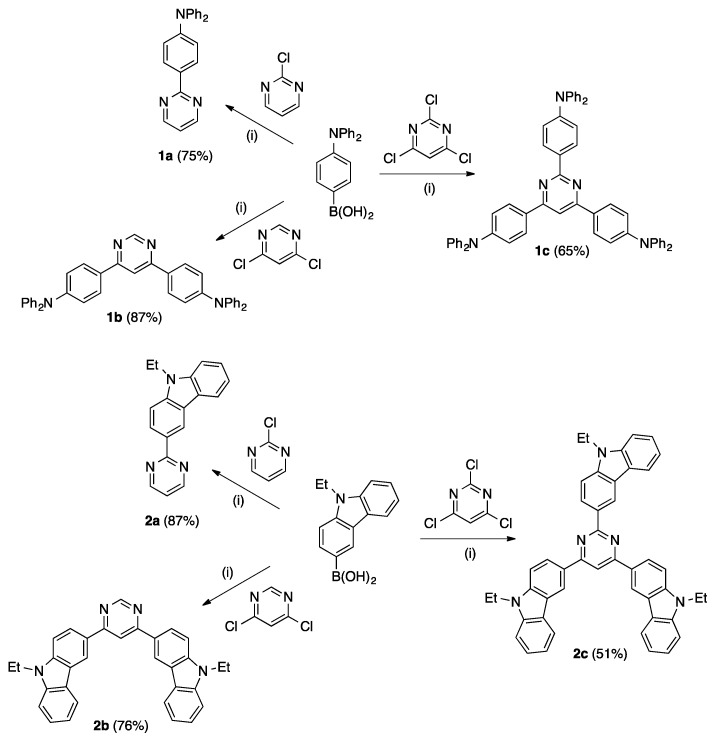
Preparation of pyrimidines **1** and **2**. (i) Toluene, Pd(PPh_3_)_4_, Na_2_CO_3_ aq., EtOH, Δ, 15 h.

Compounds **1** and **2** were obtained in moderate to good yield by Suzuki cross-coupling reaction from the corresponding chloropyrimidines and boronic acids according to a known methodology for similar pyrimidine structures (Scheme 1) [21,22,23,24]. All compounds were identified by ^1^H and ^13^C-NMR, and all previously unknown molecules were also characterized by high resolution mass spectroscopy (HRMS).

### 2.2. Photophysical Properties in Solution

**Figure 1:**
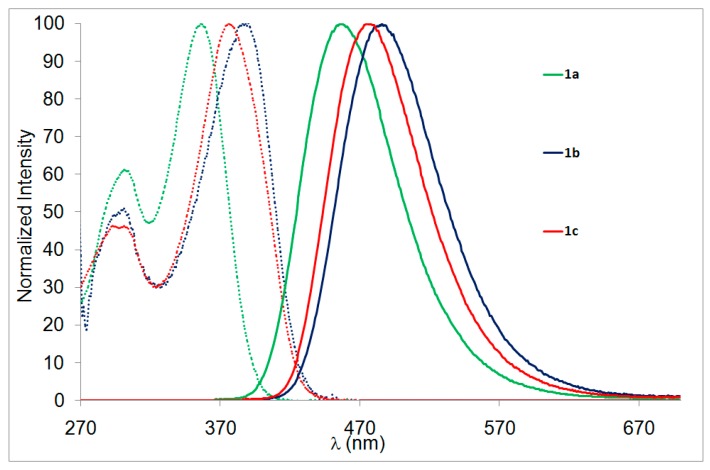
Normalized absorption (dashed lines) and emission (solid lines) spectra of compounds **1a** (green), **1b** (blue) and **1c** (red) in dichloromethane solution.

The UV-Vis and photoluminescence (PL) spectroscopic data for compounds **1** and **2** in dichloromethane are presented in Table 1. The analyses were carried out using low concentration solutions (1.0–2.0 × 10^−5^ M). Self-absorption effects were not observed under the conditions employed. As representative examples, the spectra of compounds **1a**–**c** are shown in Figure 1 (see Figure S1 in the Supporting Information for spectra of compounds **2a**–**c**).

Compounds **1**, which contain triphenylamine substituents, exhibited red-shifted absorption and emission bands with respect to their 9-ethylcarbazole analogues **2**. This finding indicates that the 9-ethyl-9*H*-carbazol-3-yl fragment is a weaker electron-donating group than triphenylamine. Triphenylamine derivatives **1** also displayed higher fluorescence quantum yields (up to 0.86 for **1b**). The 4,6-disubstituted pyrimidines **1b** and **2b** showed a red-shifted emission and an enhanced fluorescence quantum yield in comparison with their C2-monosubstituted analogues **1a** and **2a**. In contrast, the addition of a third substituent (2,4,6-triarylpyrimidines) led to a slight blue shift in the emission and a decrease in the quantum yield. A similar phenomenon has previously been observed in tristyrylpyrimidines and was attributed to a decrease in the electron-withdrawing character of the pyrimidine central core due to the C2 electron-donating substituent, which decreases the ICT along the C4 and C6 arms [32].

**Figure 2:**
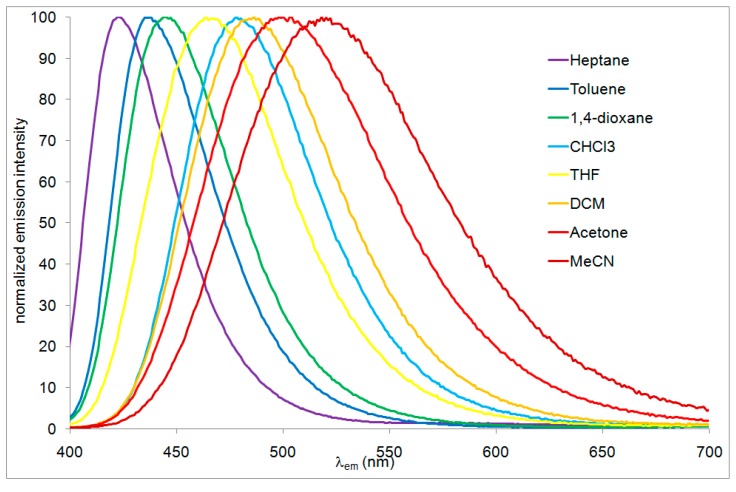
Normalized emission spectra of **1b** in different aprotic solvents.

In an effort to gain further insights into the photophysical properties of these push-pull molecules, in particular to evaluate the ICT upon excitation, the emission behaviour of compounds **1** and **2** was studied in a variety of different aprotic solvents. The data obtained are summarized in Table 2. The position of the longest wavelength absorption maximum was not affected significantly but an increase in the solvent polarity, estimated by the Dimroth–Reichardt polarity parameters [46,47], resulted in a red-shifted emission (see Figure 2 for compound **1b** and Figures S2–S6 in the Supporting Information for compounds **1a**, **1c**, and **2a**–**c**). This bathochromic shift in the emission band is consistent with stabilization of the highly polar emitting excited state by polar solvents. The solvatochromic shift of the emission band can be used to evaluate the ICT upon excitation. For all compounds, the emission maxima were plotted versus the Dimroth–Reichardt polarity parameter, and in all cases good linearity was found (see Figure S7 in the Supporting Information). The slopes of the corresponding regression lines indicate a stronger ICT for triphenylamine derivatives **1** with respect to their 9-ethylcarbazole analogues **2**. In the triphenylamine series, the slope increased in the order **1a** < **1c** < **1b**, thus indicating that the strongest ICT was obtained for the 4,6-disubstituted pyrimidine **1b**. In a similar way, the slope increased in the order **2a** < **2c** < **2b** for the 9-ethylcarbazole derivatives.

**Figure 3:**
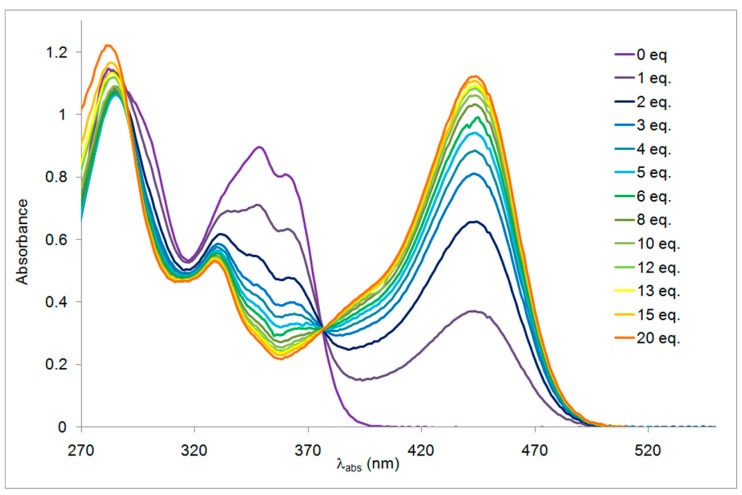
Changes in the absorption spectra of a dichloromethane solution of **2b** (*c* = 1.87 × 10^−5^ M) upon addition of CSA (0–20 equivalents).

**Figure 4:**
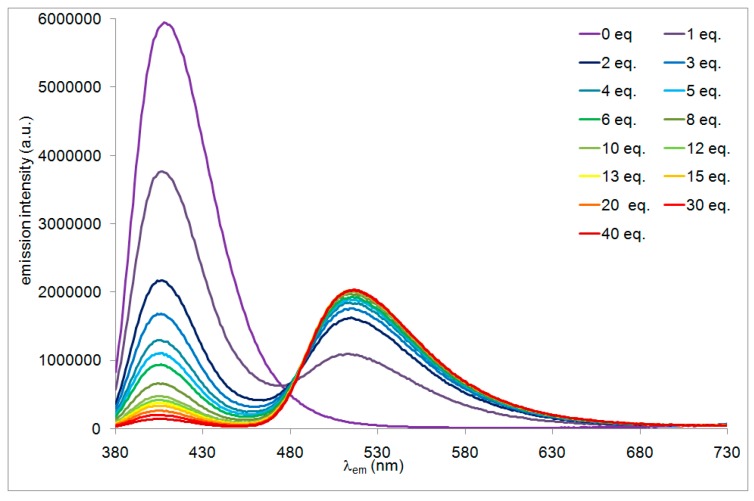
Changes in the emission spectra of a dichloromethane solution of **2b** (*c* = 1.97 × 10^−5^ M) upon addition of CSA (0–40 equivalents), λ_exc_ = 370 nm.

Photophysical measurements were also performed on a 10^−2^ M solution of camphorsulfonic acid (CSA) in dichloromethane. The results are summarized in Table 3. As one would expect, a bathochromic shift of the charge transfer absorption band was observed for all compounds due to the protonation of the pyrimidine ring [45,48]. This was associated with a dramatic quenching of the fluorescence for the triphenylamine derivatives. As a consequence, emission bands could not be identified for compounds **1a** and **1c**, whereas for **1b** a low intensity emission in the red region was detected (λ_max_ = 651 nm, Φ_F_ < 0.01). It is worth noting that the protonated form of **1b** was more emissive in chloroform solution (λ_abs_ = 493 nm, λ_em_ = 625 nm, Φ_F_ = 0.11). In contrast, the 9-ethylcarbazole derivatives **2b** and **2c** remained fluorescent and they emitted green-yellow light with high emission quantum yields (Φ_F_ = 0.63 and 0.45, respectively). The decay lifetimes (τ) were determined to be 3.6 ns and 4.2 ns (τ values for the neutral molecules were 1.8 ns and 1.7 ns, respectively). Surprisingly, **2a** was not emissive in acidified dichloromethane. The effect of protonation was studied in a more detailed way by titration of solutions of compounds **1b**, **2b** and **2c** with CSA. The changes observed in the UV-vis and emission spectra for **2b** are illustrated in Figure 3 and Figure 4, respectively (see the Supporting Information for data for compounds **1b** and **2c**). The absorption spectra showed the progressive disappearance of the charge transfer absorption band of the neutral form, whereas a red-shifted charge transfer absorption band for the protonated form progressively appeared. The presence of an isosbestic point is evident and this is characteristic of an equilibrium between two species (Figure 3). The same trend was observed in the emission spectra: the addition of CSA led to the progressive disappearance of the emission band of the neutral form and this was associated with the enhancement of a new red-shifted band corresponding to the emission of the protonated form with an isoemissive point (Figure 4).

**Figure 5:**
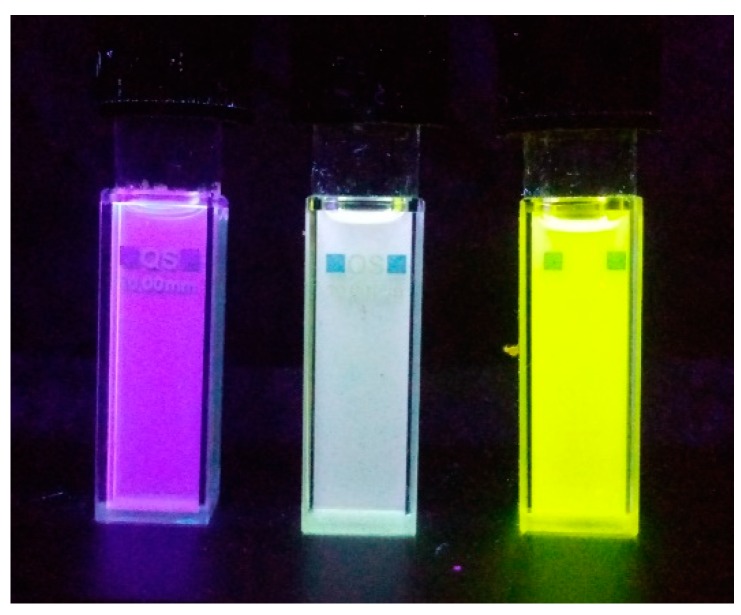
Changes in the color of a dichloromethane solution of **2c** (*c* = 1.25 × 10^−5^ M) after the addition of 45 equivalents (middle) and in 10^−2^ M CSA (right). Photographs were taken in the dark upon irradiation with a hand-held UV lamp (λ_em_ = 366 nm).

The coexistence of both neutral and protonated species with complementary emitting colors in the solution enabled white light emission to be achieved under UV-irradiation. Thus, compound **2c** emitted violet light at λ_max_ = 398 nm and this turned to green-yellow at λ_max_ = 552 nm upon protonation. Excitation at 370 nm led to the observation of white light after the addition of 45 equivalents of CSA to a 1.25 × 10^−5^ M solution of **2c** in dichloromethane (Figure 5). The same phenomenon was also observed for compounds **1b** and **2b** (Figures S11 and S12 of the Supporting Information, respectively). For **1b** and **2c**, the calculated CIE chromaticity coordinates (Table 4) were close to those of pure white light (0.33, 0.33).

### 2.3. Photophysical Properties in Solid State

**Figure 6:**
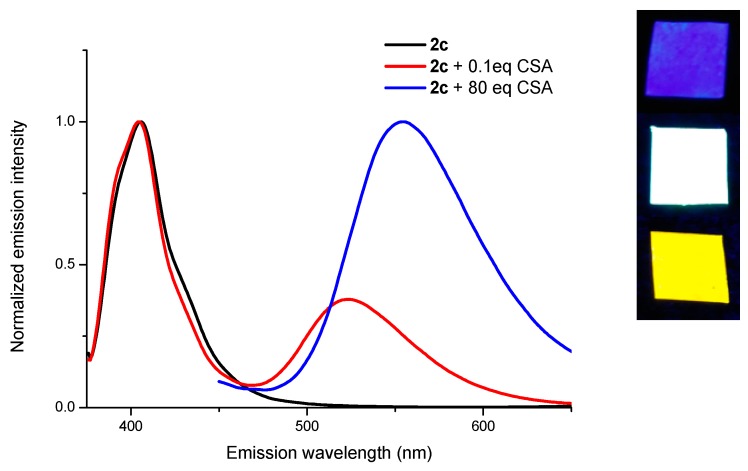
Fluorescence spectra (λ_exc_ = 365 nm) and changes in the colour of filter paper samples after immersion into a dichloromethane solution of polystyrene doped with **2c** (1 wt%) in the absence (top) and presence of 0.1 equivalents (middle) and 80 equivalents (bottom) of CSA. Photographs were taken in the dark upon irradiation with a hand-held UV lamp (λ_em_ = 366 nm).

Filter paper pieces covered with **2b** and **2c** in the absence and presence of different amounts of CSA were prepared by immersion of the filter paper into a dichloromethane solution of the appropriate compound (1 wt% doped on polystyrene). The samples were dried in air. The fluorescence spectra of the samples were acquired and emission maxima in the violet region at λ_max_ = 405 nm and 406 nm, respectively, were determined in the absence of acid. The intensities of these bands gradually decreased on increasing the amount of CSA, whereas a distinctly novel enhanced emission appeared in the green-yellow region (λ_max_ = 544 nm for **2b** and λ_max_ = 554 nm for **2c**). After careful tuning of the number of equivalents of CSA, white light was observed due to the simultaneous emission from both the neutral and protonated compounds. These significant emission changes were easily followed by the naked eye under UV irradiation (Figure 6 and Figure S13 in the Supporting Information). Energy transfer between neutral and protonated molecules has been suggested for related systems [49].

## 3. Materials and Methods

### 3.1. General Information

All solvents were reagent grade for synthesis and spectroscopic grade for photophysical measurements. The starting materials were purchased from Sigma-Aldrich (St Louis, MO, USA) or Alfa Aesar (Haverhill, MA, USA) and were used without further purification. For air- and moisture-sensitive reactions, all glassware was flame-dried and cooled under nitrogen. NMR spectra were recorded in CDCl_3_ on a Bruker Avance 300 spectrometer (^1^H at 300 MHz and ^13^C at 75 MHz, Billerica, MA, USA). The chemical shifts δ are reported in ppm and are referenced to the residual protons of the deuterated solvent or carbon nuclei (^1^H, δ = 7.27 ppm; ^13^C, δ = 77.0 ppm). The coupling constants *J* are given in Hz. In the ^1^H-NMR spectra, the following abbreviations are used to describe the peak patterns: s (singlet), d (doublet), dd (doublet of doublets), t (triplet), m (multiplet). In the ^13^C-NMR spectra, the nature of the carbons (C, CH, CH_2_ or CH_3_) was determined by performing a JMOD experiment. Melting points (°C) were measured on a Kofler hot-stage with a precision of 2 degrees (±2 °C). High-resolution mass analyses were performed at the ‘Centre Régional de Mesures Physiques de l’Ouest’ (CRMPO, Université de Rennes 1, Rennes, France) using a Bruker MicroTOF-Q II apparatus. Analytical thin layer chromatography (TLC) was performed on 60 F254 silica gel plates (Merck, Darmstadt, Germany), and compounds were visualized by irradiation with UV light at 254 and 365 nm. Flash chromatography was performed using silica SI 60 (60–200 mesh ASTM, Acros, Waltham, MA, USA). UV-visible and fluorescence spectroscopy studies were conducted on a Spex Fluoromax-3 spectrophotometer (Jobin-Yvon Horiba, Kyoto, Japan). Compounds were excited at their absorption maxima (band of lowest energy) to record the emission spectra. All solutions were measured with optical densities below 0.1. Fluorescence quantum yields (±10%) were determined relative to 9,10-bis(phenylethynyl)anthracene in cyclohexane (Φ_F_ = 1.00). Stokes shifts were calculated considering the lowest energetic absorption band.

### 3.2. General Procedure for Suzuki Cross-coupling Reactions

A stirred mixture of the chloropyrimidine derivative (1 mmol), arylboronic acid (1.2 mmol per chlorine atom), Pd(PPh_3_)_4_ (0.05 mmol per chlorine atom), 1 M aqueous sodium carbonate (1.2 mmol, 1.2 mL per chlorine atom), and ethanol (1.5 mL) in degassed toluene (15 mL) was heated at reflux under nitrogen for 15 h in a Schlenk tube. The reaction mixture was cooled, filtered, and dissolved with a mixture of AcOEt and water 1:1 (50 mL) and the organic layer was separated. The aqueous layer was extracted with AcOEt (2 × 25 mL). The combined organic extracts were dried with MgSO_4_ and the solvents were evaporated.

*2-(4-Diphenylaminophenyl)pyrimidine* (**1a**). The title compound was obtained according to the general procedure and purified by column chromatography (SiO_2_, AcOEt/petroleum ether, 3:7). Beige solid. Yield: 75% (241 mg). Mp: 173–174 °C (lit.: 168–169 °C) [50]. ^1^H-NMR (300 MHz, CDCl_3_): δ 7.17–7.05 (m, 9H), 7.31–7.27 (m, 4H), 8.28 (d, 2H, *J* = 9.0 Hz), 8.74 (d, 2H, *J* = 4.8 Hz). ^13^C-NMR (75 MHz, CDCl_3_): δ 118.2, 122.1, 123.6, 125.2, 129.2, 129.4, 130.9, 147.3, 150.3, 157.1, 164.5.

*4,6-bis(4-Diphenylaminophenyl)pyrimidine* (**1b**). The title compound was obtained according to the general procedure and purified by column chromatography (SiO_2_, AcOEt/petroleum ether, 3:7). Yellow solid. Yield: 87% (492 mg). Mp: 230–231 °C. ^1^H-NMR (300 MHz, CDCl_3_): δ 7.17–7.07 (m, 16H), 7.33–7.27 (m, 8H), 7.93 (d, 1H, *J* = 1.2 Hz), 7.99 (d, 4H, *J* = 9.0 Hz), 9.17 (d, 1H, *J* = 1.2 Hz). ^13^C-NMR (75 MHz, CDCl_3_): δ 110.9, 122.0, 123.9, 125.3, 128.1, 129.5, 129.9, 147.1, 150.4, 159.0, 163.7. HRMS (ESI/ASAP), *m*/*z* calculated for C_40_H_31_N_4_ [M + H]^+^ 567.2543, found 567.2546.

*2,4,6-tris(4-Diphenylaminophenyl)pyrimidine* (**1c**). The title compound was obtained according to the general procedure and purified by column chromatography (SiO_2_, AcOEt/petroleum ether, 3:7). Yellow solid. Yield: 65% (526 mg). Mp: >260 °C. ^1^H-NMR (300 MHz, CDCl_3_): δ 7.11–7.06 (m, 6H), 7.18–7.15 (m, 18H), 7.32–7.27 (m, 12H), 7.78 (s, 1H), 8.11 (d, 4H, *J* = 8.7 Hz), 8.51 (d, 2H, *J* = 8.7 Hz). ^13^C-NMR (75 MHz, CDCl_3_): δ 107.9, 122.2, 122.4, 123.3, 123.7, 124.9, 125.2, 128.1, 129.3, 129.4, 130.8, 132.1, 147.2, 147.4, 149.9, 150.2, 163.7, 164.0 HRMS (ESI/ASAP), *m*/*z* calculated for C_58_H_44_N_5_ [M + H]^+^ 810.3591, found 810.3591.

*2-(9-Ethyl-9H-carbazol-3-yl)pyrimidine* (**2a**). The title compound was obtained according to the general procedure and purified by column chromatography (SiO_2_, AcOEt/petroleum ether, 3:7). Beige solid. Yield: 87% (238 mg). Mp: 136–137 °C. ^1^H-NMR (300 MHz, CDCl_3_): δ 1.47 (t, 3H, *J* = 7.2 Hz), 4.41 (q, 6H, *J* = 7.2 Hz), 7.14 (t, 1H, *J* = 4.8 Hz), 7.30–7.27 (m, 1H), 7.52–7.42 (m, 3H), 8.22 (d, 1H, *J* = 7.8 Hz), 8.61 (d, 1H, *J* = 7.8 Hz), 8.82 (d, 2H, *J* = 5.1 Hz), 9.22 (s, 2H). ^13^C-NMR (75 MHz, CDCl_3_): δ 13.9, 37.7, 108.4, 108.7, 118.1, 119.5, 120.8, 121.0, 123.3, 123.5, 126.0, 126.2, 128.6, 140.6, 141.8, 157.2, 165.6. HRMS (ESI/ASAP), *m*/*z* calculated for C_18_H_16_N_3_ [M + H]^+^ 274.1338, found 274.1343.

*4,6-bis(9-Ethyl-9H-carbazol-3-yl)pyrimidine* (**2b**). The title compound was obtained according to the general procedure and purified by column chromatography (SiO_2_, AcOEt/petroleum ether, 1:1). Pale yellow solid. Yield: 76% (352 mg). Mp: 170–171 °C (lit.: 176–177 °C) [45]. ^1^H-NMR (300 MHz, CDCl_3_): δ 1.45 (t, 6H, *J* = 6.9 Hz), 4.33 (q, 4H, *J* = 6.9 Hz), 7.53–7.30 (m, 8H), 8.32–8.24 (m, 5H), 8.99 (s, 2H), 9.36 (s, 1H). ^13^C-NMR (75 MHz, CDCl_3_): δ 13.8, 37.7, 108.6, 108.8, 111.5, 119.6, 119.7, 120.8, 123.2, 123.5, 124.9, 126.2, 127.9, 140.6, 141.6, 159.0, 164.8.

*2,4,6-tris(9-Ethyl-9H-carbazol-3-yl)pyrimidine* (**2c**). The title compound was obtained according to the general procedure and purified by column chromatography (SiO_2_, AcOEt/petroleum ether, 3:7) followed by recrystallization from CH_2_Cl_2_/*n*-heptane. Pale yellow solid. Yield: 51% (330 mg). Mp: 176–177 °C. ^1^H-NMR (300 MHz, CDCl_3_): δ 1.52 (t, 9H, *J* = 6.3 Hz), 4.46 (q, 6H, *J* = 7.2 Hz), 7.37–7.33 (m, 3H), 7.62–7.47 (m, 9H), 8.22 (d, 1H, *J* = 2.4 Hz), 8.38–8.33 (m, 3H), 8.59 (2H, d, *J* = 8.7 Hz), 9.04 (1H, dd, *J*_1_ = 8.7 Hz, *J*_2_ = 1.2 Hz), 9.14 (s, 2H), 9.59 (s, 1H). ^13^C-NMR (75 MHz, CDCl_3_): δ 13.9, 37.8, 108.2, 108.7, 108.8, 119.2, 119.4, 119.8, 120.8, 120.9, 121.2, 123.2, 123.4, 123.5, 123.8, 125.4, 125.7, 126.1, 126.8, 129.1, 130.0, 140.6, 141.6. HRMS (ESI/ASAP), *m*/*z* calculated for C_46_H_38_N_5_ [M + H]^+^ 660.3122, found 660.3122.

## 4. Conclusions

Push-pull pyrimidines substituted with a different number of either triphenylamine or 9-ethylcarbazole groups were prepared by Suzuki cross-coupling reaction from the corresponding chloropyrimidines and boronic acids. The molecules presented absorption wavelengths in the UV region and emitted violet-blue light in dichloromethane solution with a higher fluorescence quantum yield and a stronger ICT observed for the triphenylamine derivatives. The addition of acid was accompanied by a dramatic quenching of the fluorescence except for the 9-ethylcarbazole derivatives **2b** and **2c** (and partially for **1b**). In these cases, protonation led to the progressive disappearance of the emission band and the appearance of a new red-shifted complementary emitting band. Thus, white photoluminescence could be obtained by controlled protonation. White emission was also achieved in solid state.

## Figures and Tables

**Table 1:** UV-Vis and photoluminescence (PL) data for compounds **1** and **2** in CH_2_Cl_2_ solution.

Compd	λ_abs_ (nm)	ε (mM^−1^ cm^−1^)	λ_em_ (nm)	Φ_F_	Stokes Shift (cm^−1^)
**1a**	356, 301	21.7, 13.3	456	0.83	6160
**1b**	388, 301	52.0, 26.4	486	0.86	5200
**1c**	376, 299, 293	76.6, 36.7, 36.9	475	0.52	5540
**2a**	323, 284, 276 (sh)	22.6, 51.6, 33.8	382	0.32	4780
**2b**	360, 348, 284	43.2, 48.2, 50.1	407	0.77	3210
**2c**	367 (sh) 339, 299, 282 (sh)	20.4, 69.2, 84.2, 64.1	398	0.35	2120

**Table 2:** Emission solvatochromism of compounds **1** and **2** in various aprotic solvents.

Compd	*n*-Heptane 30.9 *^a^*	Toluene 33.9 *^a^*	1,4-Dioxane 36.0 *^a^*	THF 37.4 *^a^*	CHCl_3_ 39.1 *^a^*	CH_2_Cl_2_ 40.7 *^a^*	Acetone 42.2 *^a^*	MeCN 45.6 *^a^*
**1a**	406	420	429	449	450	456	462	477
**1b**	418	437	444	464	478	486	498	518
**1c**	413	432	438	466	466	475	492	508
**2a**	354/373	361/378	362/379	379	383	382	382	389
**2b**	371	383	384	395	406	407	413	424
**2c**	379	384	386	385	398	398	407	415

*^a^* ET(30) Dimroth–Reichardt polarity parameter in kcal mol^−1^.

**Table 3:** UV/Vis and PL data for compounds **1** and **2** in acid solution (10^−2^ M CSA in CH_2_Cl_2_).

Compd	λ_abs_ (nm)	ε (mM^−1^ cm^−1^)	λ_em_ (nm)	Φ_F_	Stokes Shift (cm^−1^)
**1a**	424, 373 (sh), 268	17.2, 9.3, 30.5	-	-	-
**1b**	488	48.3	651	<0.01	5130
**1c**	486, 375, 274	68.2, 30.4, 88.5	-	-	-
**2a**	396	27.3	-	-	-
**2b**	444, 390, 285	63.9, 28.0, 64.3	519	0.63	3250
**2c**	443, 330	41.9, 29.0	552	0.45	4460

**Table 4:** CIE coordinates for compounds **1** and **2** in solution.

Compd	Chromaticity Coordinates (x, y)		
	Neutral Form	Protonated Form	Mixture of Neutral and Protonated Forms
**1a** *^a^*	(0.15, 0.15)	-	-
**1b** *^b^*	(0.18, 0.33)	(0.62, 0.37)	(0.32, 0.34) *^c^*
**1c** *^a^*	(0.16, 0.25)	-	-
**2a** *^a^*	(0.18, 0.04)	-	-
**2b** *^a^*	(0.16, 0.03)	(0.29, 0.59)	(0.24, 0.39) *^d^*
**2c** *^a^*	(0.17, 0.04)	(0.42, 0.56)	(0.30, 0.34) *^e^*

*^a^* In dichloromethane solution (*c* = 1.0–2.0 × 10^−5^ M). *^b^* In chloroform solution (*c* = 9.76 × 10^−6^ M). *^c^* 50 equivalents of CSA, λ_exc_ = 400 nm. *^d^* 2 equivalents of CSA, λ_exc_ = 370 nm. *^e^* 45 equivalents of CSA, λ_exc_ = 370 nm.

## Supplementary Materials

The following material is available online: Figure S1: absorption and emission spectra of compounds **2a**–**c** in dichloromethane solution; Figures S2–S6: emission spectra of **1a**, **1c**, and **2a**–**c** in different aprotic solvents; Figure S7: emission maxima as a function of the Dimroth–Reichardt polarity parameter E_T_(30) for compounds **1** and **2**; Figures S8–S10: changes in the absorption and emission spectra of a solution of **1b** and **2b** upon addition of CSA; Figures S11 and S12: changes in the color of a solution of **1b** and **2b** after the addition of CSA; Figure S13: fluorescence spectra and changes in the color of **2b** in solid state after the addition of CSA; Figures S14–S23: ^1^H, ^13^C-NMR and HRMS spectra of compounds **1** and **2**.
