# Supplementary material for: Carbazole- and Triphenylamine-Substituted Pyrimidines: Synthesis and Photophysical Properties
Source: Molecules. 2019 May 5;24(9):1742. doi: 10.3390/molecules24091742 (PMC6540165; doi:10.3390/molecules24091742)
Supplement: Supplementary file 1 [file molecules-24-01742-s001.pdf]

Supporting Information

Carbazole- and Triphenylamine-Substituted Pyrimidines: Synthesis and Photophysical Properties

Sylvain Achelle,*[,1] Julián Rodríguez-López,*[,2] Massinissa Larbani,[1]
Rodrigo Plaza-Pedroche,[2] Françoise Robin-le Guen[1]

[1] Université de Rennes, CNRS, Institut des Sciences Chimiques de Rennes - UMR 6226, F 35000 Rennes, France

[2] Área de Química Orgánica, Facultad de Ciencias y Tecnologías Químicas, Universidad de Castilla-La-Mancha, Avda. Camillo José Cela 10, 13071 Ciudad Real, Spain

Absorption and emission spectra of compounds **2a-c** in dichloromethane solution.......S2

Emission spectra of **1a** in different aprotic solvents..S2

Emission spectra of **1c** and **2a** in different aprotic solvents...S3

Emission spectra of **2b** and **2c** in different aprotic solvents S4

Emission maxima (λ_{em}) as a function of the Dimroth-Reichardt polarity parameter $E_T(30)$ for compounds **1** and **2** S5

Changes in the absorption and emission spectra of a chloroform solution of **1b** upon addition of CSA...S6

Changes in the emission spectra of a dichloromethane solution of **2b** upon addition of CSA..S7

Changes in the colour of a chloroform solution of **1b** after the addition of CSA...........S7

Changes in the colour of a dichloromethane solution of **2b** after the addition of CSA ..S8

Fluorescence spectra and changes in the colour of **2b** in solid state after the addition of CSA S8

^{1}H and ^{13}C NMR spectra of **1a**...S9

^{1}H and ^{13}C NMR and HRMS spectra of **1b** S10

^{1}H and ^{13}C NMR and HRMS spectra of **1c**...S11

^{1}H and ^{13}C NMR and HRMS spectra of **2a**...S12

^{1}H and ^{13}C NMR spectra of **2b**..S13

^{1}H and ^{13}C NMR and HRMS spectra of **2c**...S14

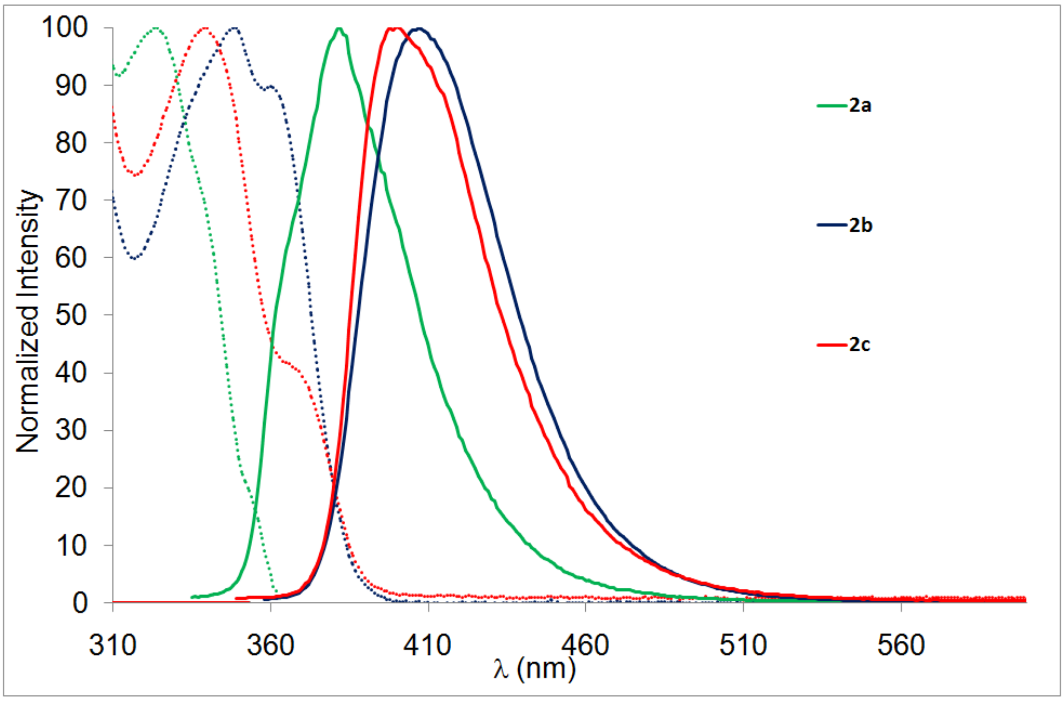

Figure S1. Normalized absorption (dashed lines) and emission (solid lines) spectra of compounds **2a** (green), **2b** (blue) and **2c** (red) in dichloromethane solution.

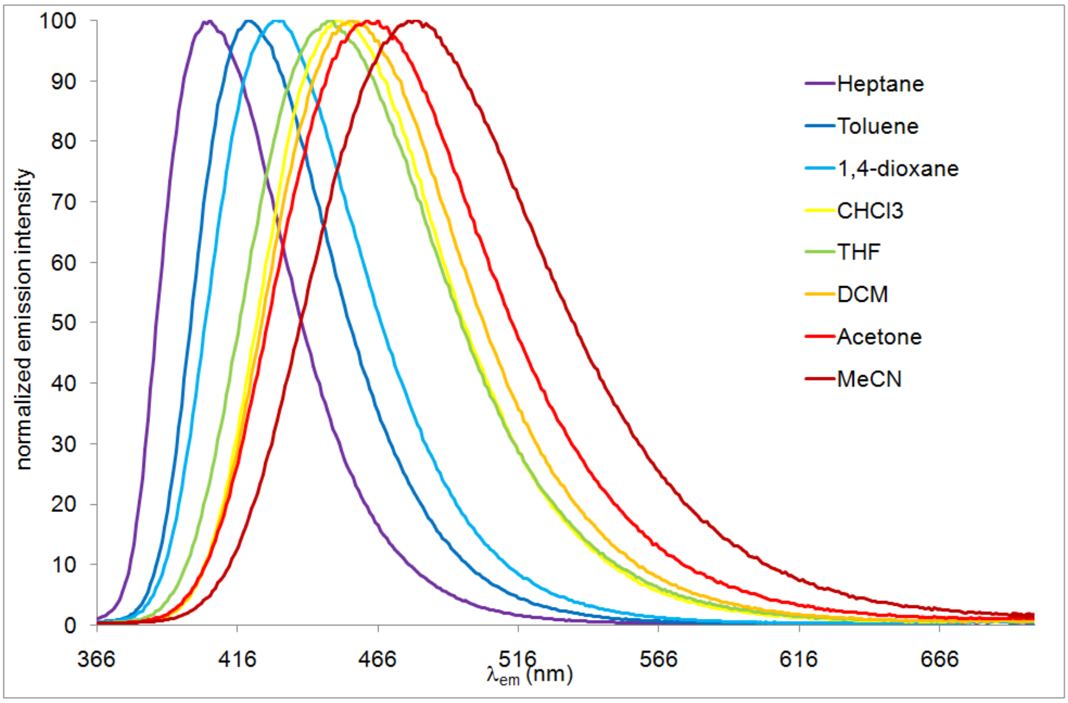

Figure S2. Normalized emission spectra of **1a** in different aprotic solvents.

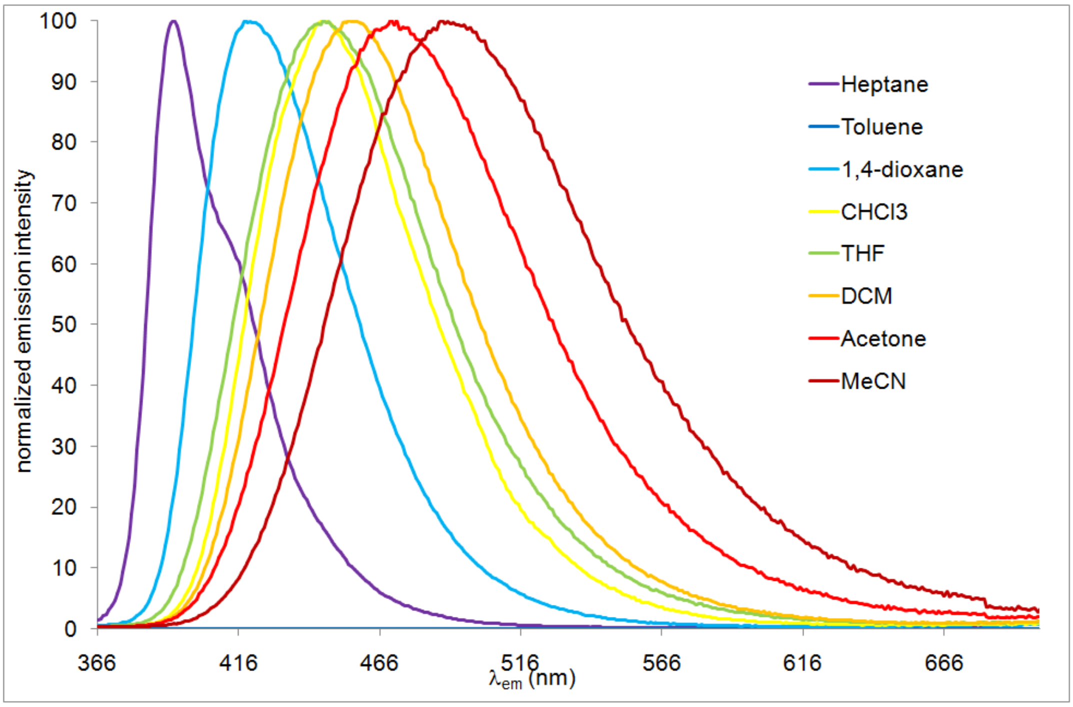

Figure S3. Normalized emission spectra of **1c** in different aprotic solvents.

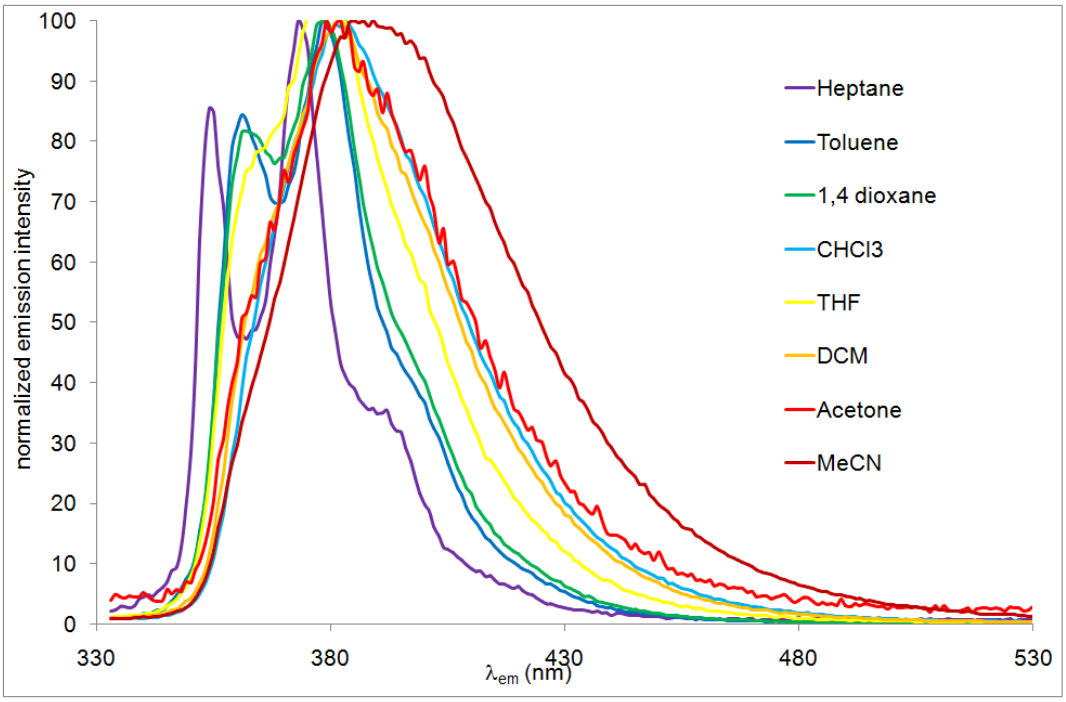

Figure S4. Normalized emission spectra of **2a** in different aprotic solvents.

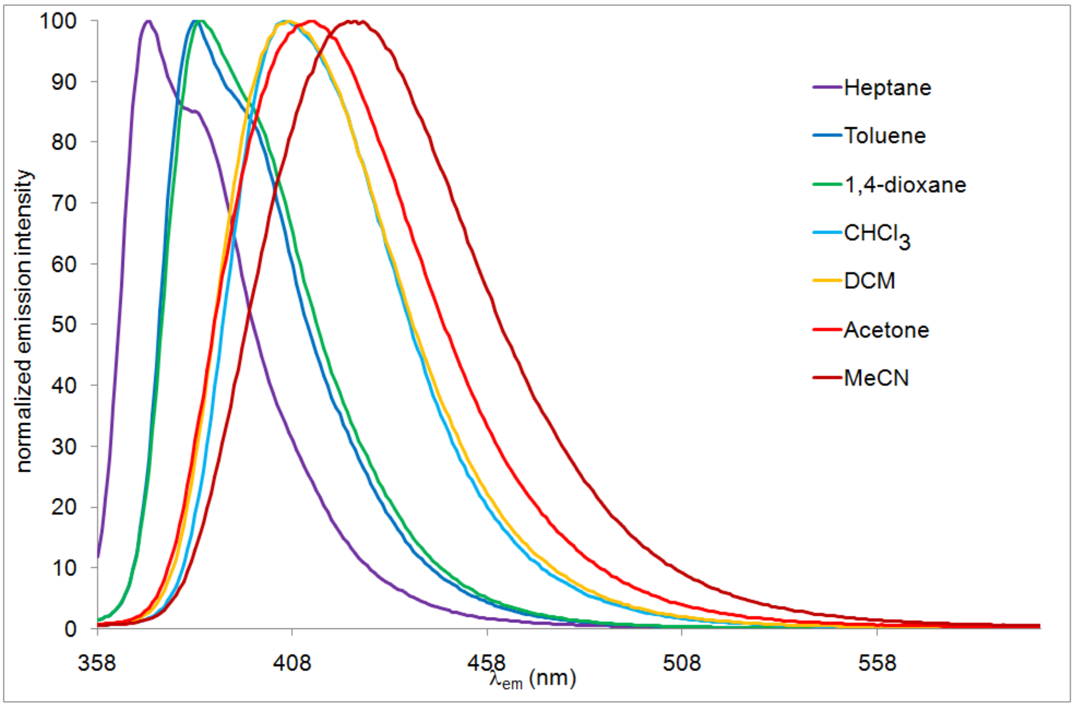

Figure S5. Normalized emission spectra of **2b** in different aprotic solvents.

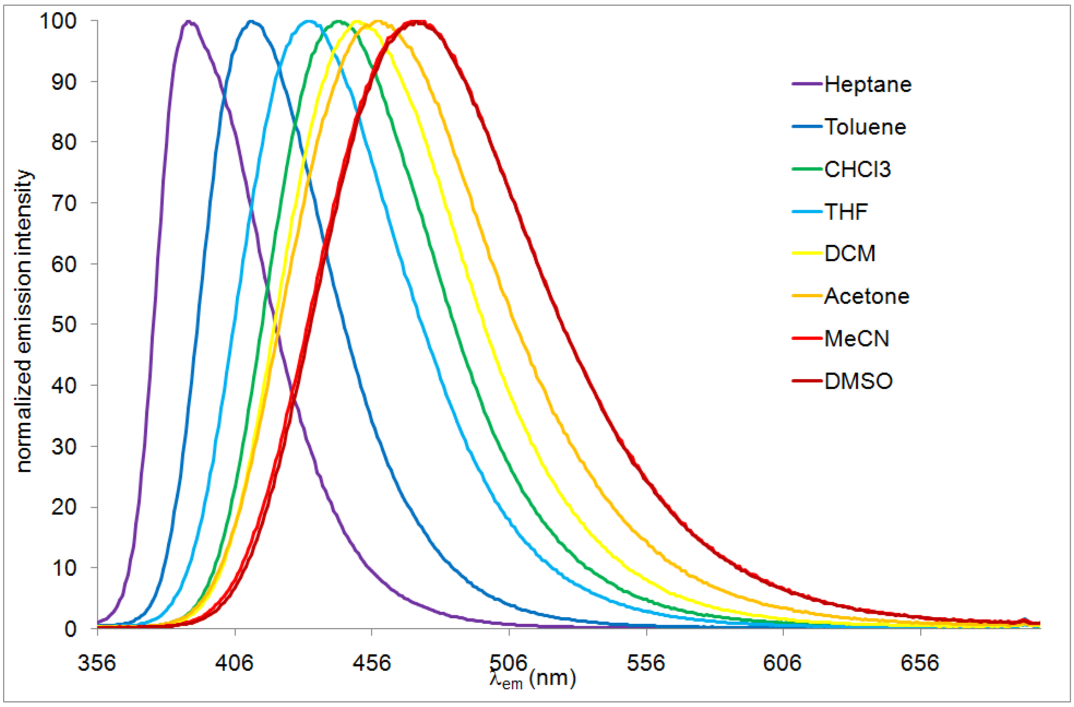

Figure S6. Normalized emission spectra of **2c** in different aprotic solvents.

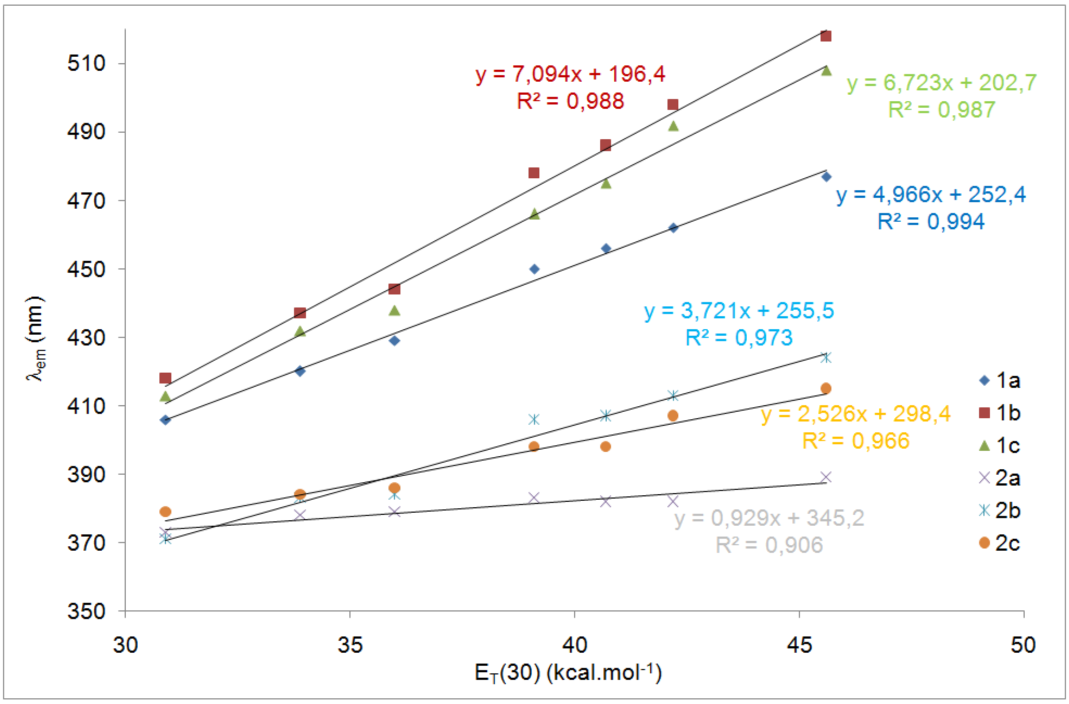

Figure S7. Emission maxima (λ_{em}) as a function of the Dimroth-Reichardt polarity parameter $E_T(30)$ for compounds **1** and **2**.

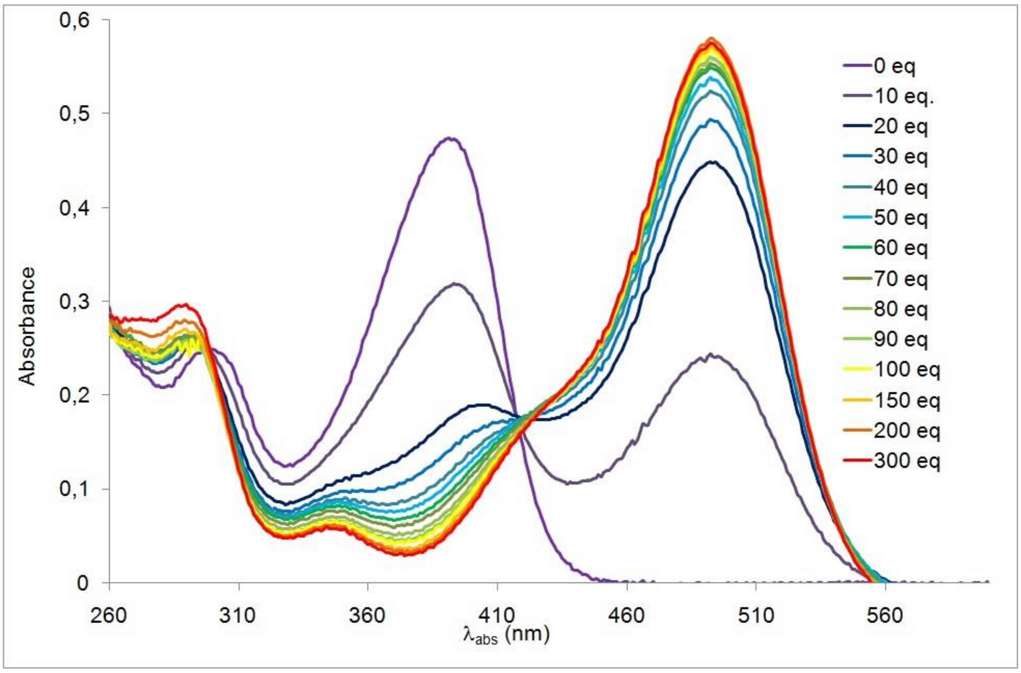

Figure S8. Changes in the absorption spectra of a chloroform solution of **1b** (c = 9.76 × 10^{-6} M) upon addition of CSA (0-300 equivalents).

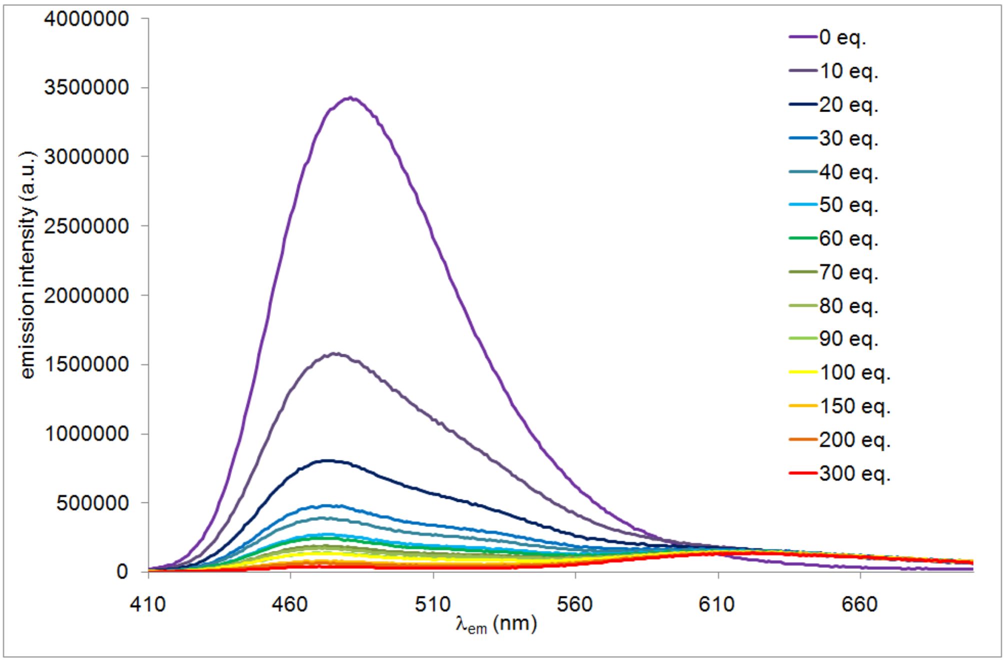

Figure S9. Changes in the emission spectra of a chloroform solution of **1b** (c = 9.76 × 10^{-6} M) upon addition of CSA (0-300 equivalents). λ_{exc} = 400 nm.

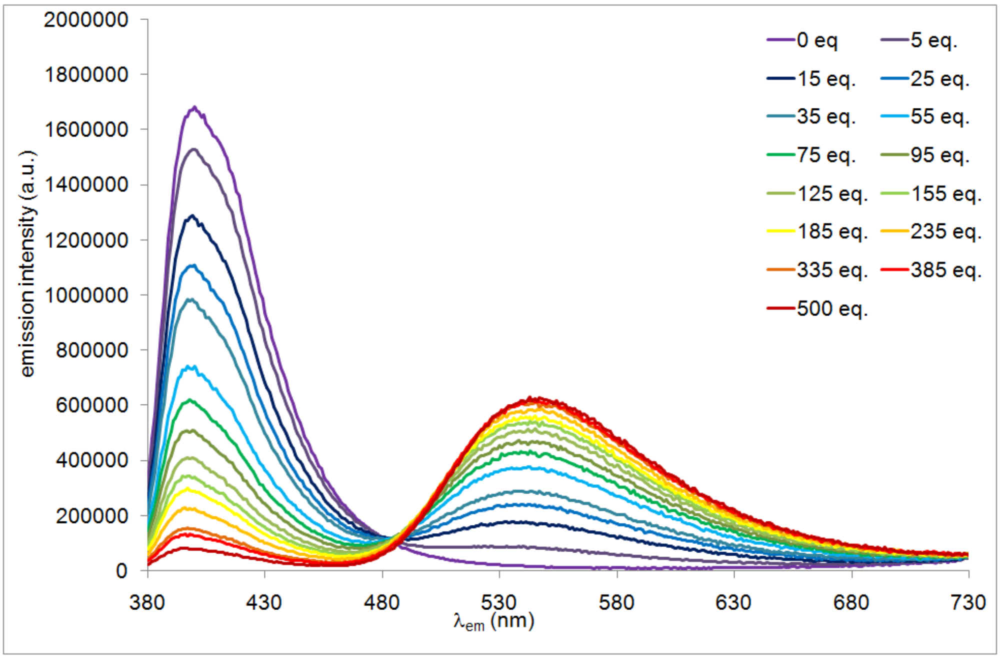

Figure S10. Changes in the emission spectra of a dichloromethane solution of **2c** (c = 1.25 × 10^{-5} M) upon addition of CSA (0-500 equivalents). λ_{exc} = 370 nm.

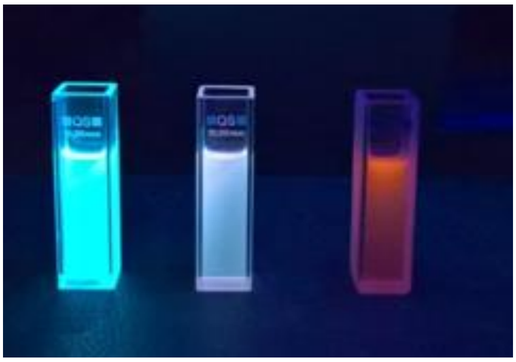

Figure S11. Changes in the colour of a chloroform solution of **1b** (c = 9.76 × 10^{-6} M) after the addition of 30 equivalents (middle) and in 10^{-2} M CSA (right). Photographs were taken in the dark upon irradiation with a hand-held UV lamp (λ_{em} = 366 nm).

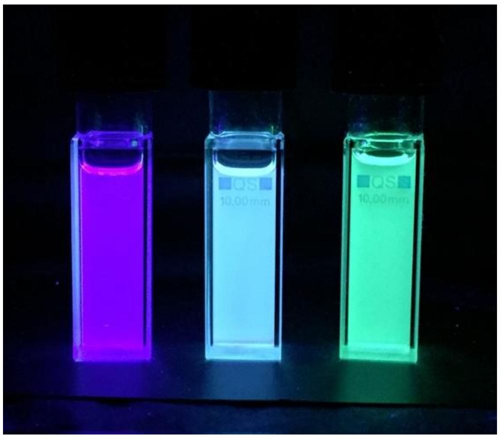

Figure S12. Changes in the colour of a dichloromethane solution of **2b** (c = 1.97 × 10^{-5} M) after the addition of 2 equivalents (middle) and in 10^{-2} M CSA (right). Photographs were taken in the dark upon irradiation with a hand-held UV lamp (λ_{em} = 366 nm).

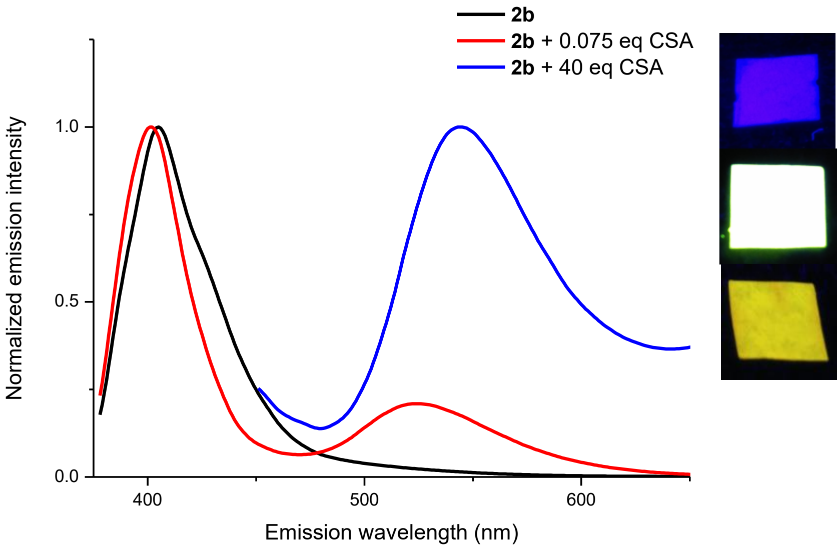

Figure S13. Fluorescence spectra (λ_{exc} = 365 nm) and changes in the colour of filter paper samples after immersion into a dichloromethane solution of polystyrene doped with **2b** (1 wt%) in the absence (top) and the presence of 0.075 equivalents (middle) and 40 equivalents (bottom) of CSA. Photographs were taken in the dark upon irradiation with a hand-held UV lamp (λ_{em} = 366 nm).

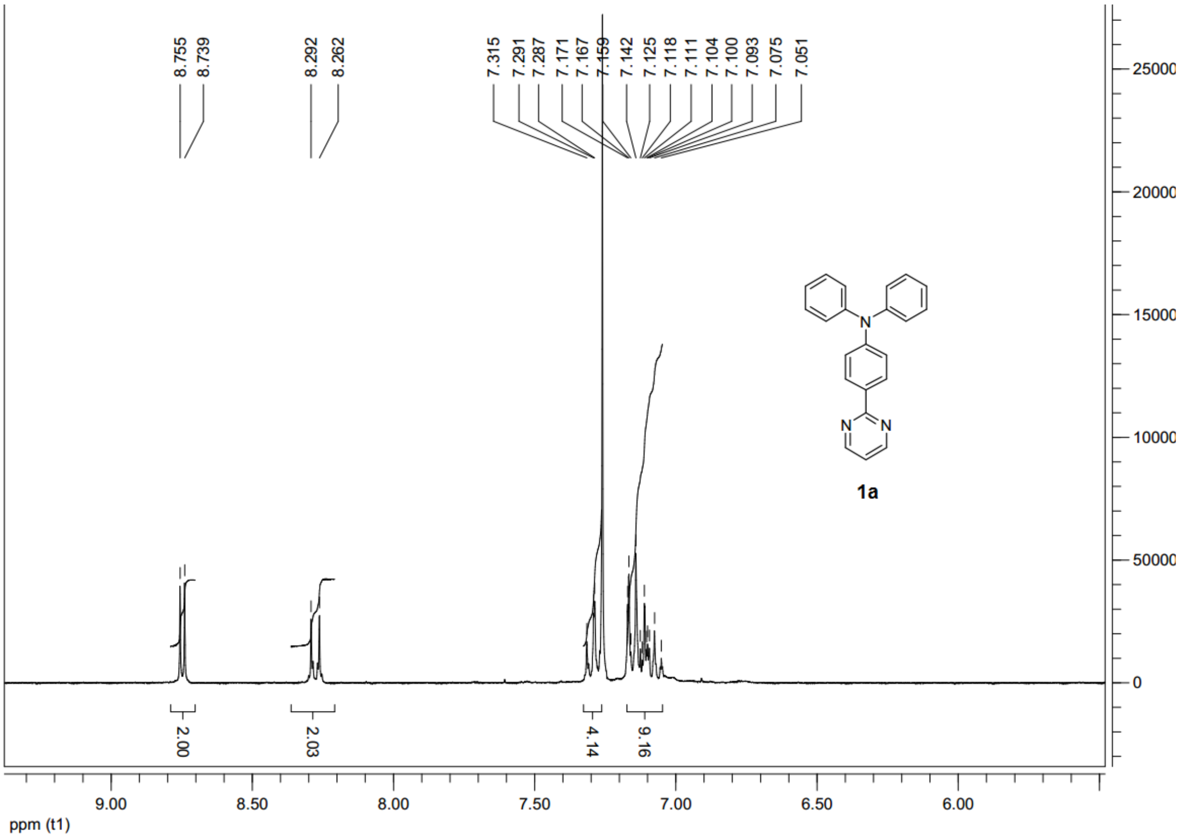

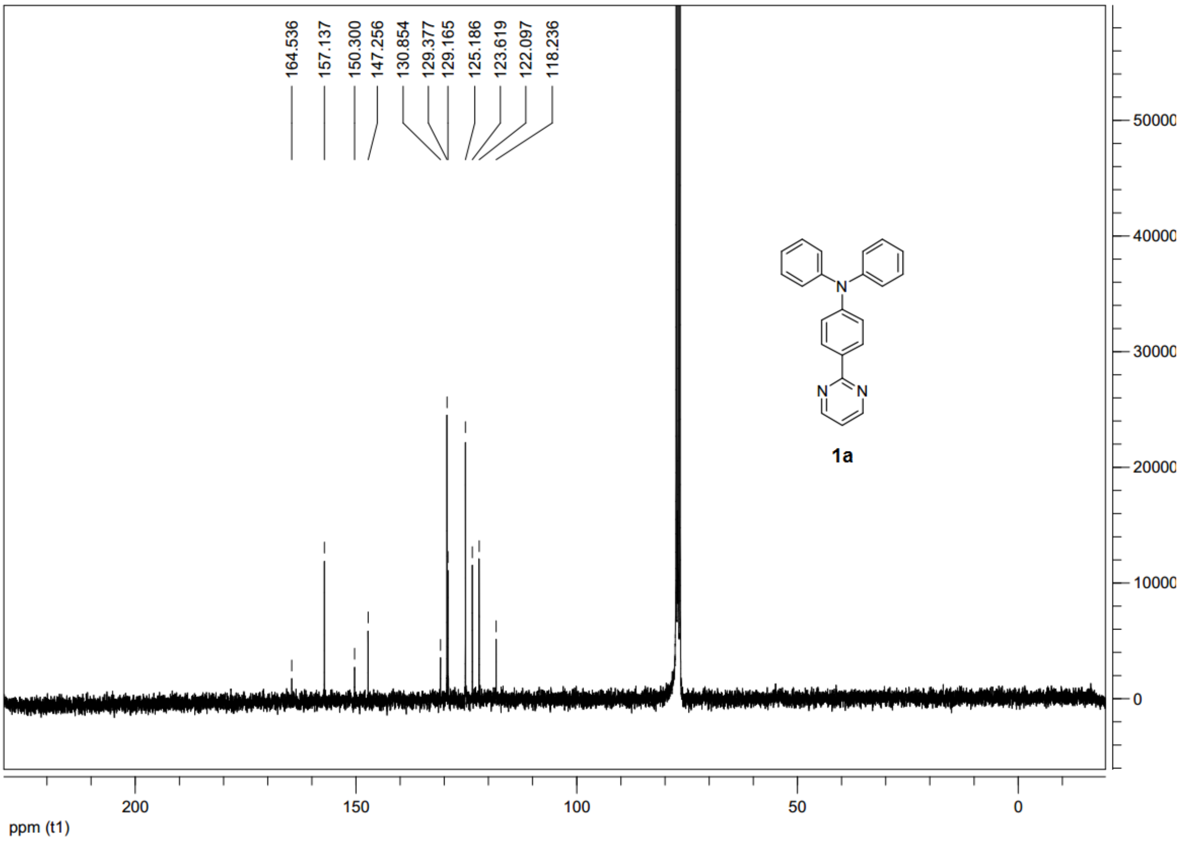

Figure S14. ^{1}H and ^{13}C NMR spectra of compound **1a** in $CDCl_3$.

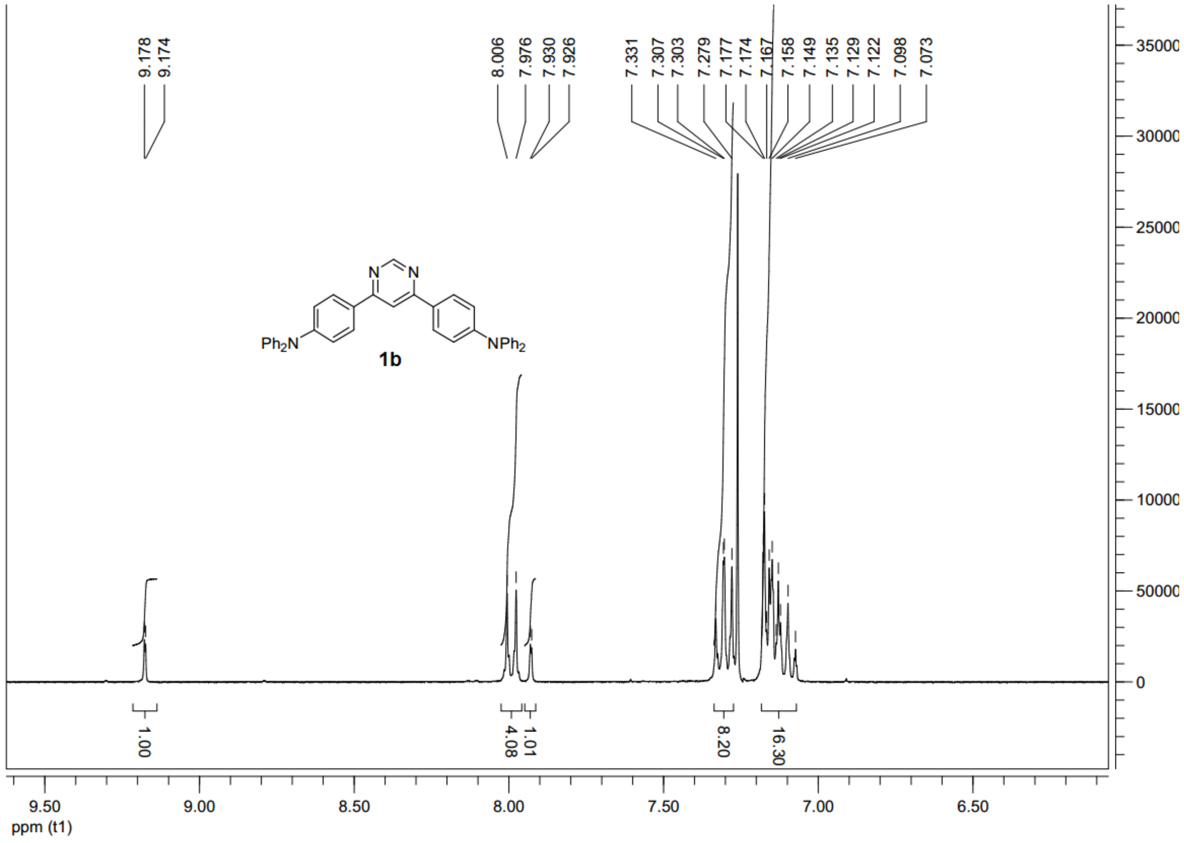

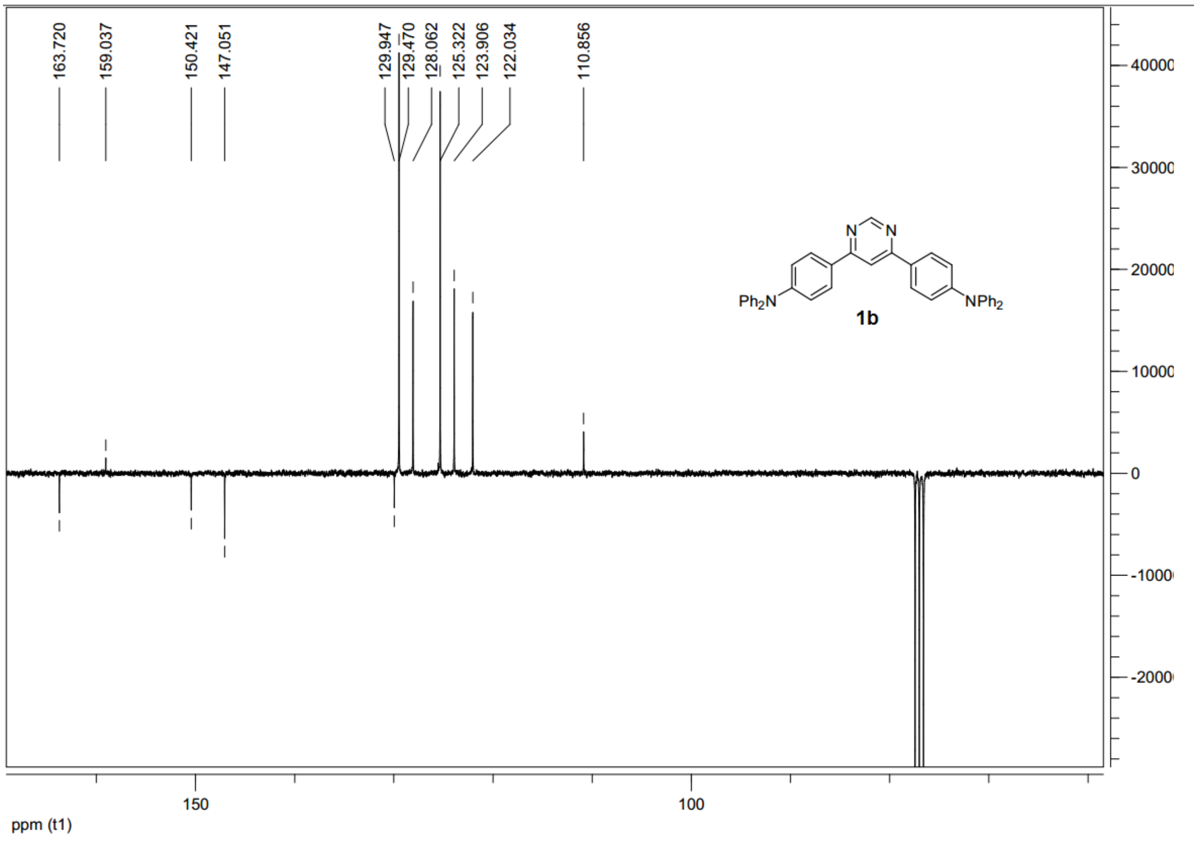

Figure S15. ^{1}H and ^{13}C NMR spectra of compound **1b** in $CDCl_3$.

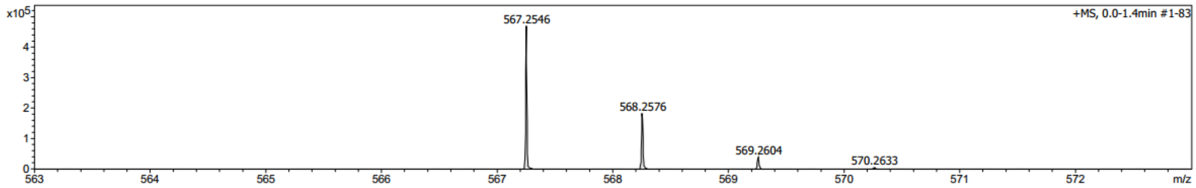

Figure S16. HRMS spectra of compound **1b**.

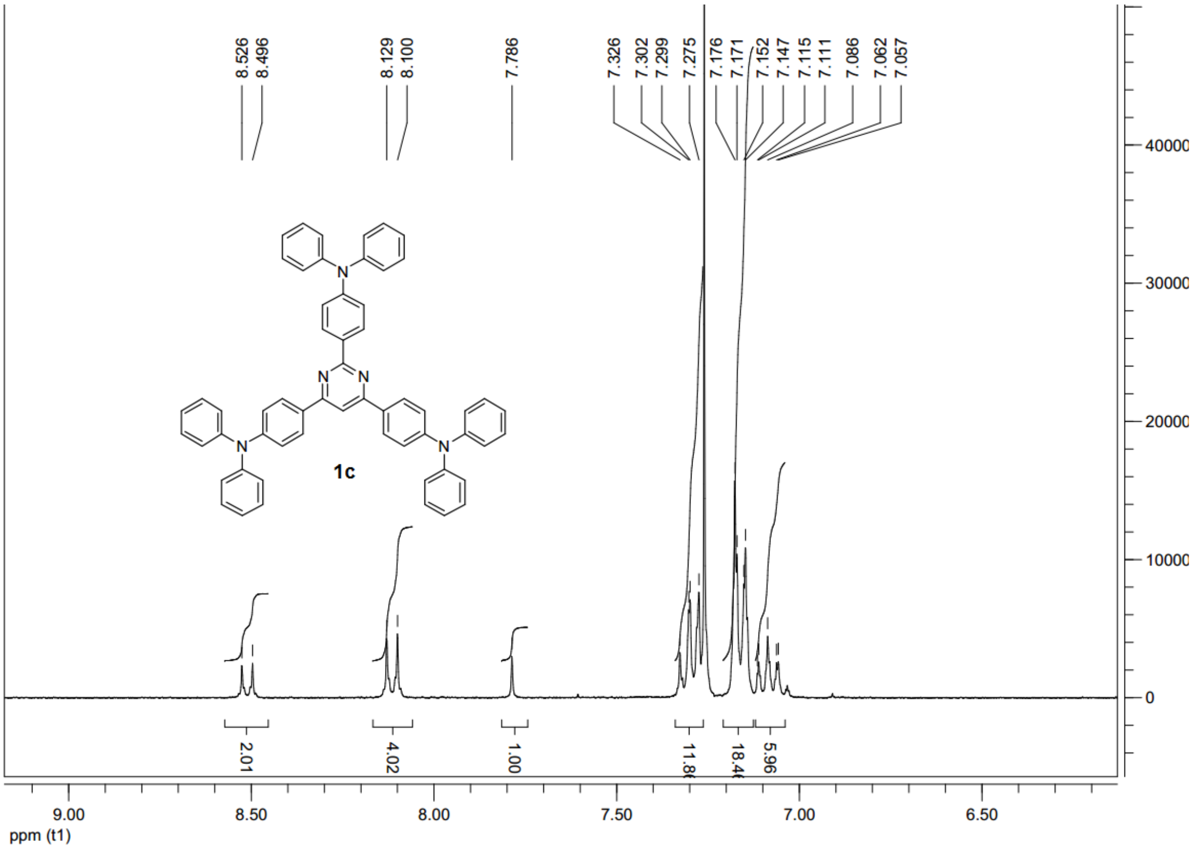

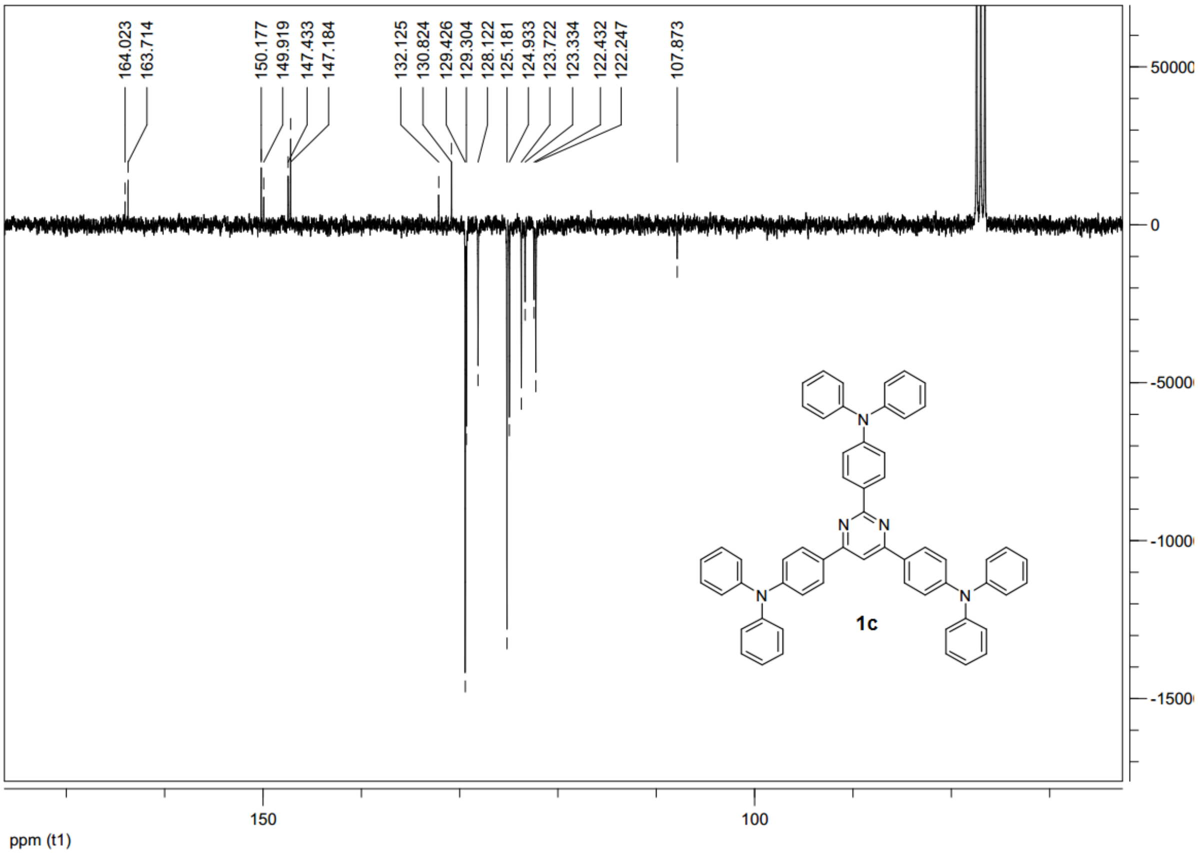

Figure S17. ^{1}H and ^{13}C NMR spectra of compound **1c** in $CDCl_3$.

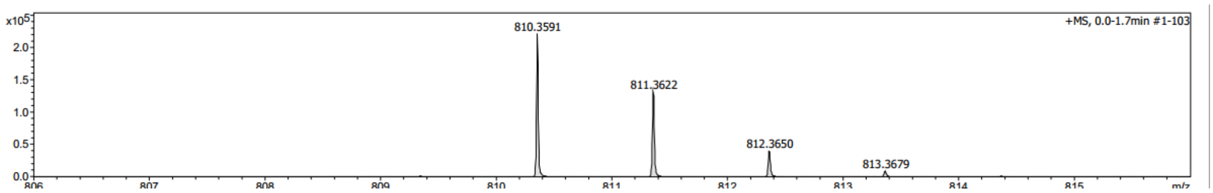

Figure S18. HRMS spectra of compound **1c**.

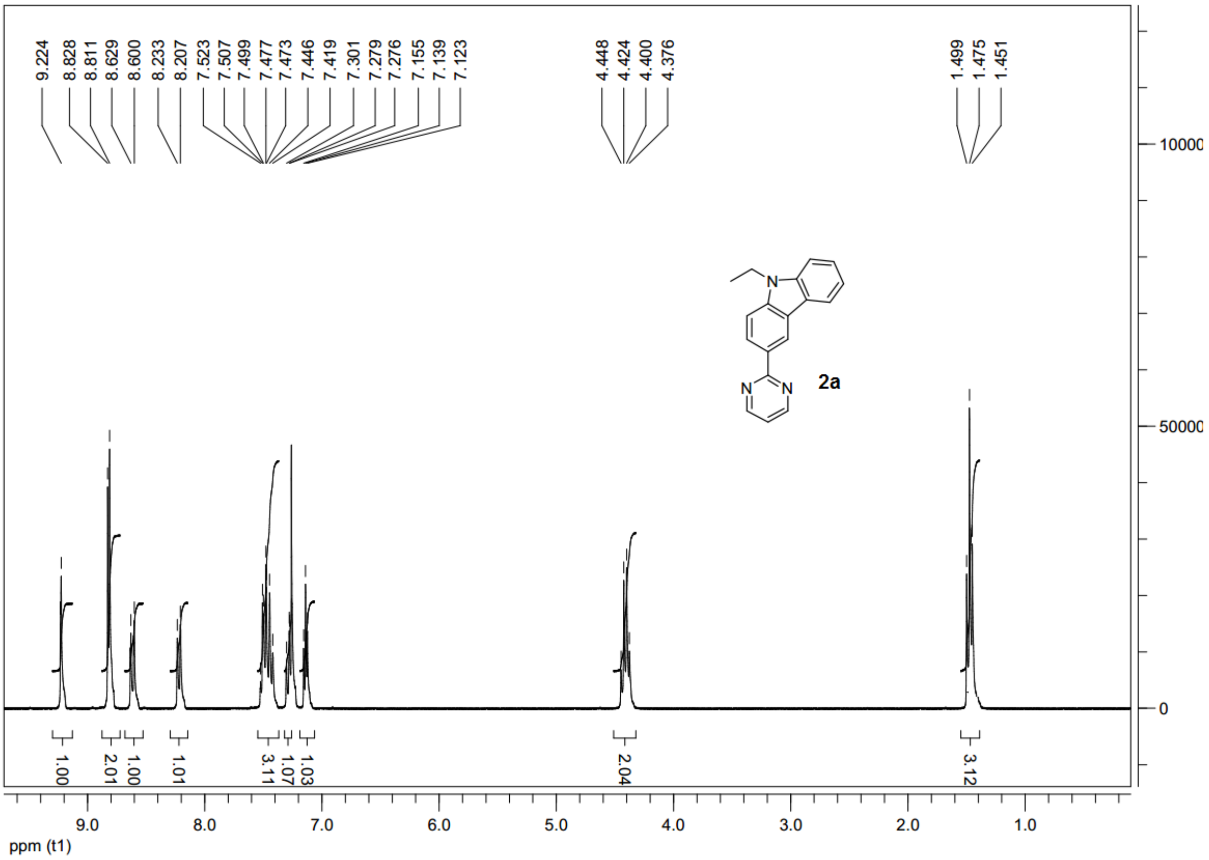

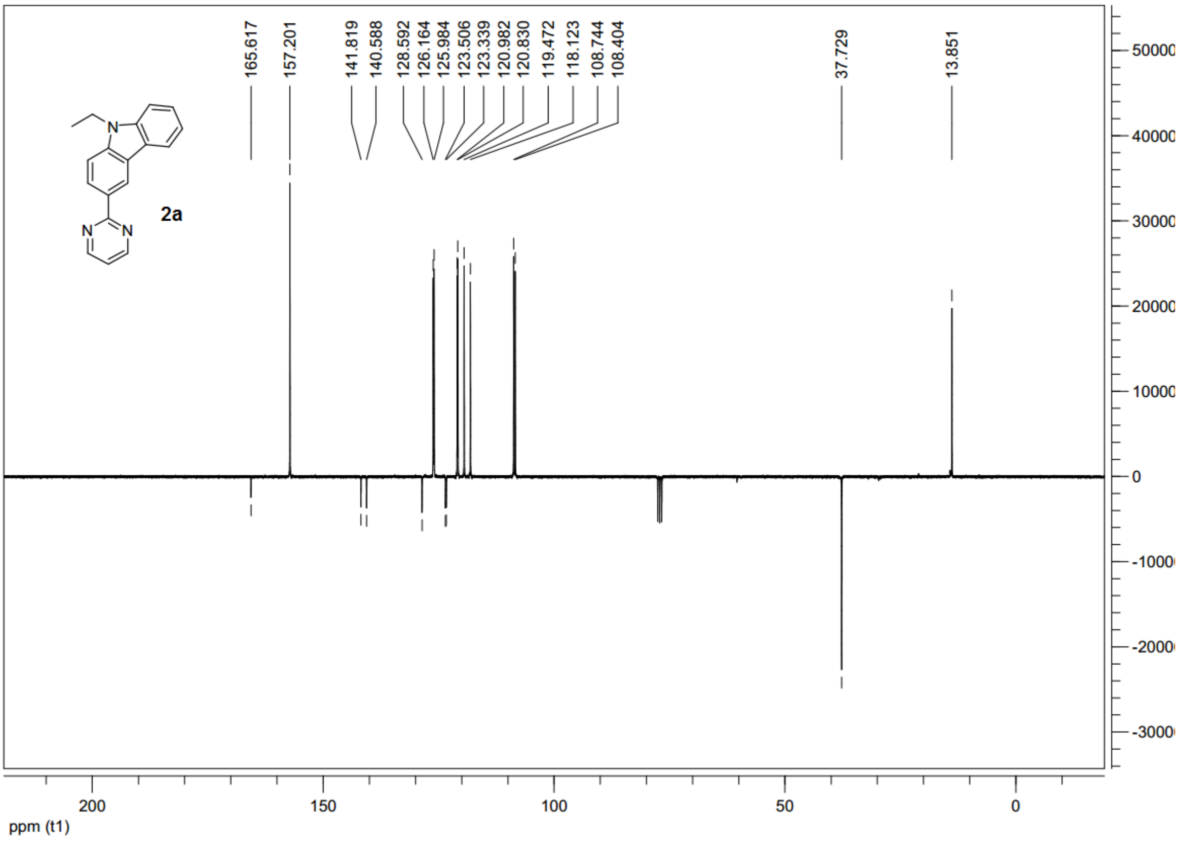

Figure S19. ^{1}H and ^{13}C NMR spectra of compound **2a** in $CDCl_3$.

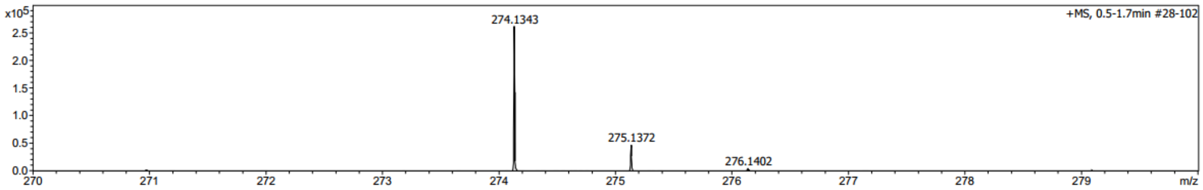

Figure S20. HRMS spectra of compound **2a**.

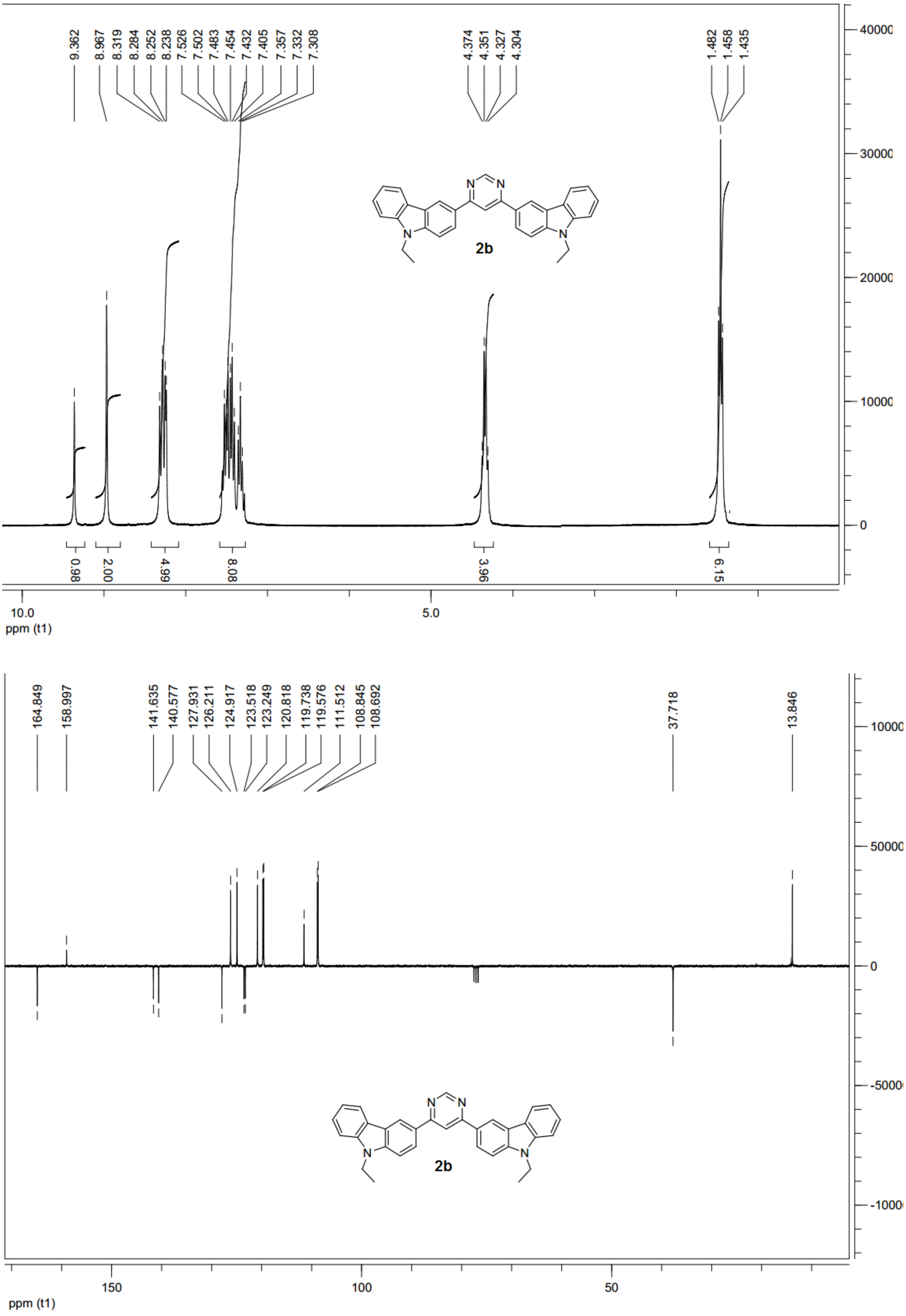

Figure S21. ^{1}H and ^{13}C NMR spectra of compound **2b** in $CDCl_3$.

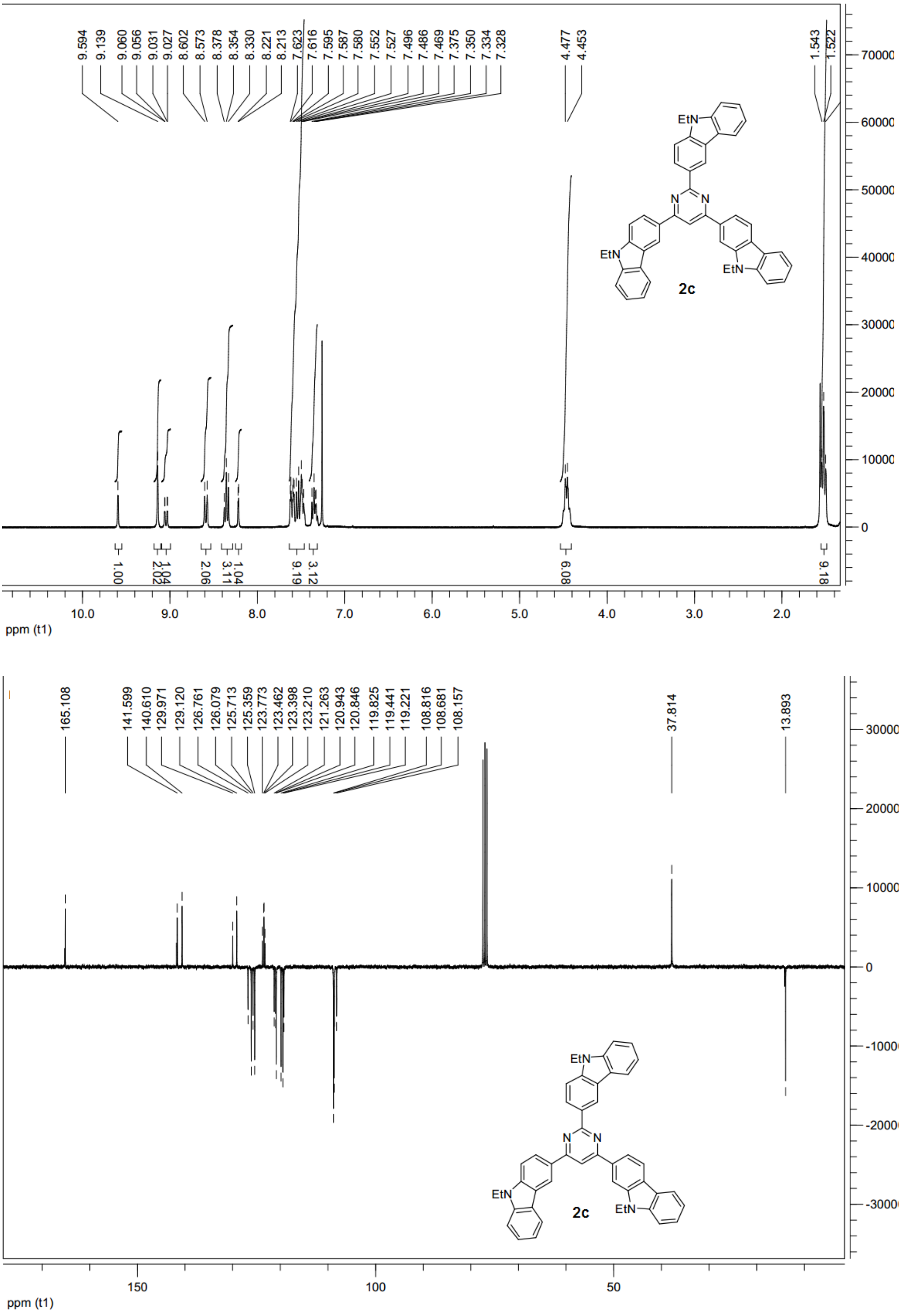

Figure S22. ^{1}H and ^{13}C NMR spectra of compound **2c** in $CDCl_3$.

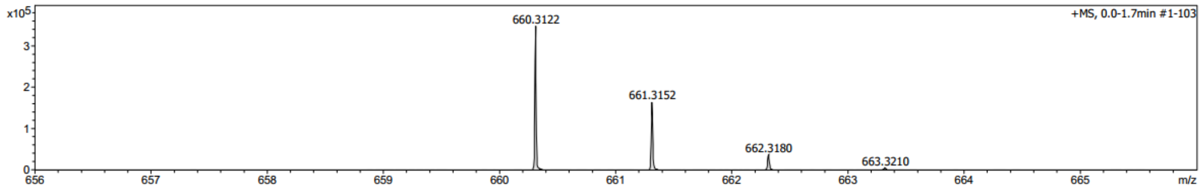

Figure S23. HRMS spectrum of compound **2c**.
